# Supplementary material for: Identification of qPCR reference genes suitable for normalising gene expression in the developing mouse embryo
Source: Wellcome Open Res. 2022 Sep 21;6:197. Originally published 2021 Aug 3. [Version 2] doi: 10.12688/wellcomeopenres.16972.2 (PMC9024131; doi:10.12688/wellcomeopenres.16972.2)
Supplement: Peer review history

# Peer review: Reviewer response for version 2

**DOI:** 10.21956/wellcomeopenres.20390.r52483

**Published:** 2022-10-05

**Authors:** Liang Hu

**Affiliations:** 1 Reproductive and Genetic Hospital of CITIC-XIANGYA, Changsha, China

No new comments.

Is the work clearly and accurately presented and does it cite the current literature?

Yes

If applicable, is the statistical analysis and its interpretation appropriate?

Yes

Are all the source data underlying the results available to ensure full reproducibility?

Yes

Is the study design appropriate and is the work technically sound?

Partly

Are the conclusions drawn adequately supported by the results?

Partly

Are sufficient details of methods and analysis provided to allow replication by others?

Yes

Reviewer Expertise:

Genetics

I confirm that I have read this submission and believe that I have an appropriate level of expertise to confirm that it is of an acceptable scientific standard.

# Peer review: Reviewer response for version 2

**DOI:** 10.21956/wellcomeopenres.20390.r52485

**Published:** 2022-09-23

**Authors:** Anush Kosakyan

**Affiliations:** 1 Institute of Parasitology, Biology Centre Czech Academy of Science, České Budějovice, Czech Republic

I am satisfied with the author's answers and corrections and do not have further comments.

Is the work clearly and accurately presented and does it cite the current literature?

Yes

If applicable, is the statistical analysis and its interpretation appropriate?

Yes

Are all the source data underlying the results available to ensure full reproducibility?

Yes

Is the study design appropriate and is the work technically sound?

Yes

Are the conclusions drawn adequately supported by the results?

Partly

Are sufficient details of methods and analysis provided to allow replication by others?

Yes

Reviewer Expertise:

Microbial eukaryotes, taxonomy, phylogeny, transcriptomics, genomics

I confirm that I have read this submission and believe that I have an appropriate level of expertise to confirm that it is of an acceptable scientific standard.

# Peer review: Reviewer response for version 1

**DOI:** 10.21956/wellcomeopenres.18735.r49745

**Published:** 2022-04-21

**Authors:** Liang Hu

**Affiliations:** 1 Reproductive and Genetic Hospital of CITIC-XIANGYA, Changsha, China

In this manuscript, the authors tried to identify the stably expressed genes as normalizing controls in E11.5-E18.5 mouse embryos for future studies. It is interesting and important for some researchers. 

 Several main problems are listed as follows:

1. The diversity and the complexity of the cell types in mouse embryos may affect the reliability of this study. In this study, the authors only chose head and forelimb samples, which cannot reflect other tissue and cell types. The best way is to analyze the published single-cell sequencing data and find the candidate genes, and then select the best ones by quantitative PCR. 
2. I would recommend the authors verified the stability of their selected genes in this study in other published sequencing data.

Is the work clearly and accurately presented and does it cite the current literature?

Yes

If applicable, is the statistical analysis and its interpretation appropriate?

Yes

Are all the source data underlying the results available to ensure full reproducibility?

Yes

Is the study design appropriate and is the work technically sound?

Partly

Are the conclusions drawn adequately supported by the results?

Partly

Are sufficient details of methods and analysis provided to allow replication by others?

Yes

Reviewer Expertise:

Genetics

I confirm that I have read this submission and believe that I have an appropriate level of expertise to confirm that it is of an acceptable scientific standard, however I have significant reservations, as outlined above.

# Response

**Published:** 2022-09-12

**Authors:** John Hildyard

We thank the reviewer for their careful consideration of our manuscript, and their helpful comments. We address these as discussed below (original reviewer comments in italics for reference).

*Several main problems are listed as follows:*

*The diversity and the complexity of the cell types in mouse embryos may affect the reliability of this study. In this study, the authors only chose head and forelimb samples, which cannot reflect other tissue and cell types. The best way is to analyze the published single-cell sequencing data and find the candidate genes, and then select the best ones by quantitative PCR.*

*I would recommend the authors verified the stability of their selected genes in this study in other published sequencing data.*

 This is an excellent point to raise: thank you. Embryos are indeed a complex, dynamic transcriptional environment, with many different cell types present. Critically, however, many of these specific nuances are lost following bulk RNA extraction, which necessarily homogenises all cells present. Our approach is intended to address the normalisation of gene expression in this latter scenario only: as it is often both more practical and more expedient to determine gene expression behaviour in bulk extracts, especially when the focus is on expression of only a few, specific targets. Moreover, study of gene expression via such approaches is markedly less expensive, placing it within the reach of more investigators. As we state within our introduction:

 “Recent technological advances have permitted elegant studies of embryonic gene expression down to single-cell level ^19^^, ^^20^ , suggesting that detailed study of the interactions between individual cell lineages might now be possible, however such approaches also represent a substantial undertaking both in time and resources. Furthermore, these approaches are not without limitation: the whole transcriptome nature of the method typically favours breadth over depth ^21^^, ^^22^ : large-scale changes involving many gene components will be mapped well, while more specific, smaller-scale processes can be missed. In particular, constraints in read depth mean low abundance transcripts can be under-represented in such datasets ^23^ , and thus more conventional methods remain vital research tools.”

 As we show, isolating specific embryonic tissues prior to RNA extraction (heads, forelimbs) does alter the transcriptional behaviour measured (presumably as a consequence of the more restricted cellular milieu present in such tissue subsets), but as we further shown in our manuscript, the reference genes identified here as appropriate for whole embryos remain applicable even within these subset isolates. We do not assume that the genes isolated will be applicable within *all* embryonic tissue subsets (we deem this unlikely, and have edited the text to make this distinction more explicit), but repeating the work presented, over the developmental stages shown, for increasingly specific (and potentially challenging to isolate) tissue subsets seems firmly beyond the scope of this study. Our work does, however, represent a solid technical and instructive foundation for investigators who might wish to pursue such further studies.

 With respect to single-cell data, we do not assume our candidate genes will necessarily apply to single-cell data, and indeed, we would strongly advise against such an approach: the depth and detail of single-cell sequencing data offers alternative, more appropriate normalisation methods that can be determined *a priori,* on a sample-by-sample basis, or even a cell-by-cell basis, if necessary (Lytal *et al*, doi: 10.3389/fgene.2020.00041). By the same token, we do not feel that single-cell data can be readily used to inform bulk cDNA normalisation: the transcriptional behaviour of individual cells and cell types is not necessarily representative of behaviour at whole tissue level.

 We also note that RNAseq data (bulk or single-cell) is not strictly analogous to the cDNA approaches used here: RNAseq most commonly uses oligodT priming (and/or oligodT purification), leading to an inherent 3’ bias. As our data shows, 3’ bias can strongly affect the measured expression of long genes. We avoid this bias by use of random priming (and demonstrate the difference this makes), but such adjustments cannot necessarily be translated to RNAseq methodologies.

 Finally, we note that others have shown that use of RNAseq datasets is not required for reference gene identification (Sampathkumar et al, doi:10.1371/journal.pcbi.1009868), and that (as the reviewer rightly notes) any genes identified as potential candidates via RNAseq would need to be validated using the methods we already employ in this manuscript. As we show, our highest scoring candidates are eminently suitable references already: we are reluctant to recreate this entire study with additional genes for (at best) a fractional gain in score.

 We hope this addresses the reviewer's concerns.

# Peer review: Reviewer response for version 1

**DOI:** 10.21956/wellcomeopenres.18735.r49742

**Published:** 2022-04-19

**Authors:** Anush Kosakyan

**Affiliations:** 1 Institute of Parasitology, Biology Centre Czech Academy of Science, České Budějovice, Czech Republic

This study validates potential reference genes for gene expression studies in the developing mouse embryo from E11.5 to E18.5. Authors investigated expression of 15 candidate genes in whole embryos, heads and forelimbs, and suggested *AP3D1*, *RPL13A* and *PAK1IP1* as the most suitable ones for normalising gene expression in the developing mouse embryo. The manuscript is overall well written and structured, the methods are transparent, however there are several points that must be considered before indexing:

**Abstract:**

 "Unexpectedly, all methods suggest that many genes within our candidate panel are acceptable references, though *AP3D1*, *RPL13A* and *PAK1IP1* are the strongest performing genes overall. *HPRT1* and *B2M* are conversely poor choices and show strong developmental regulation. We further show that normalization using our three highest-scoring references can reveal subtle patterns of developmental expression even in genes ostensibly ranked as acceptably stable ( *CDC40*, *HTATSF1*)."

- Rather than saying here that HPRT1 and B2M are conversely poor choices (in general, we are not interested in what is the poor choice, but what is the best choice), I would say which genes performed best specifically for the overall embryo level and specifically for head and forelimbs, because some future studies might focus only on head or forelimbs.

**Introduction:**

 "This panel includes candidates widely used historically ( *18S*, *GAPDH*, *ACTB*), those that we have shown be strong references in mouse skeletal muscle ( *CSNK2A2*, *AP3D1*, *RPL13A*), and also shown by others to be viable references in rodent brains ( *UBC*, *HPRT1*, *RPL13A, 18S*). To maximise the power of our study, we have assessed reference gene suitability using all four algorithms described above (geNorm, deltaCt, BestKeeper and Normfinder)."

- Provide references for 18S, GAPDH, ACTB. Also, it would be great if you could provide a table (or just an extension of Table 1) with a brief description of the condition/scenario in which the gene was suggested/validated as the best reference gene and the corresponding reference. That would make your introduction really valuable.

**Methods:**

 "Uterine horns were quickly removed and placed on ice (additional organs and muscles were collected from the adults for separate studies)."

- I did not understand why this information is relevant to this study.

"RNA yield and purity were assessed via nanodrop (ND1000) and samples with 260/230 ratios below 1.7 were subjected to a second precipitation."

- = > just for your information, the optimal values for RNA purity are: 260/280 = 2, and 260/230 = 2-2.2.

"All sequences are provided below:

 18S F 5’-GTAACCCGTTGAACCCCATT-3’

 18S R 5’-CCATCCAATCGGTAGTAGCG-3’

 HPSF F 5’-GGACTAATTATGGACAGGACTG-3’

 HPSF R 5’-GCTCTTCAGTCTGATAAAATCTAC-3’

 Dp71 F 5’-GTGAAACCCTTACAACCATGAG-3’

 Dp71 R 5’-CTTCTGGAGCCTTCTGAGC-3’"

- Make a table for all gene primer sequences and give the efficiency values for each.

**Results:**

- Fig2. I am a little confused about this figure or maybe the legend is not clear enough. Are these points in each boxplot for all samples (i.e. all biological replicates for whole embryos, heads and forelimbs) per gene? Why is C shown without a box?

**Discussion:**

- It can be seen from Fig. 2 that the Ct values for the proposed reference genes are between 22 and 23, indicating that the genes are comparatively highly expressed. I would add a few lines to discuss whether you see a limitation in using these genes for the normalization of low expressed genes.
- I would also add a few lines emphasizing that the proposed reference genes are uniformly expressed throughout embryonic development, whereas the choice of these genes might be different if we were looking at individual parts (e.g., head, forelimbs) or scenarios.

Is the work clearly and accurately presented and does it cite the current literature?

Yes

If applicable, is the statistical analysis and its interpretation appropriate?

Yes

Are all the source data underlying the results available to ensure full reproducibility?

Yes

Is the study design appropriate and is the work technically sound?

Yes

Are the conclusions drawn adequately supported by the results?

Partly

Are sufficient details of methods and analysis provided to allow replication by others?

Yes

Reviewer Expertise:

Microbial eukaryotes, taxonomy, phylogeny, transcriptomics, genomics

I confirm that I have read this submission and believe that I have an appropriate level of expertise to confirm that it is of an acceptable scientific standard, however I have significant reservations, as outlined above.

# Response

**Published:** 2022-09-12

**Authors:** John Hildyard

We thank the reviewer for their careful and detailed assessment of our manuscript, and their insightful suggestions (which we discuss below -where appropriate, the reviewer's comments are reiterated for easy reference, in italics).

*Rather than saying here that HPRT1 and B2M are conversely poor choices (in general, we are not interested in what is the poor choice, but what is the best choice), I would say which genes performed best specifically for the overall embryo level and specifically for head and forelimbs, because some future studies might focus only on head or forelimbs.*

 Thank you for this suggestion. We feel for studies of this nature it is important not only to identify strong candidates, but also to highlight those that are not simply ‘less strong’ candidates, but that are actively poor candidates (such as genes that exhibit strong developmental regulation: HPRT1 and B2M). Use of these genes as references in embryonic contexts runs the risk of generating data that is not necessarily poorly normalised (i.e. high variance, low statistical value) but *incorrectly* normalised (i.e. low variance, statistically tractable, but not reflective of biological reality). We feel this latter point is worth emphasising, especially when such genes might perform strongly under other contexts -we and others have shown that HPRT1 is a very strong candidate when normalising muscle gene expression, for example (Hildyard *et al*: DOI: 10.3233/JND-170267, Wang *et al*: DOI: 10.3892/mmr.2019.10102).

 We have, however, edited the abstract to better illustrate the overall suitability of our strongest candidates: as we note in our discussion, the three genes identified *are* the strongest performing in whole embryos and in heads alone (“In embryos, heads, or all samples together, *AP3D1* is the strongest candidate, while *RPL13A*, *PAK1IP1* and *CSNK2A2* are also highly ranked”), and while 18S and EIF4A scored fractionally higher for use in forelimbs, forelimb data normalised using these two were highly comparable to data normalised using AP3D1, RPL13A and PAK1IP1, suggesting that these latter three do indeed represent a universal panel (we provide extended data to specifically illustrate this). The edited abstract is now as follows (edited text in bold):

 “Unexpectedly, all methods suggest that many genes within our candidate panel are acceptable references, though *AP3D1*, *RPL13A* and *PAK1IP1* are the strongest performing genes overall **(scoring highly in whole embryos, heads or forelimbs alone, and in all samples collectively)**.”

*Provide references for 18S, GAPDH, ACTB.*

 An entirely reasonable suggestion: thank you. As we note, these three are widely used historically: so widely, in fact, that it might almost be easier to provide citations that do *not* use one or more of these three. While the observation that these three are not optimal under all circumstances (and are in fact often markedly poor choices) is gradually gaining acceptance, use of these genes is still prevalent throughout the literature. We have thus provided citations of review articles addressing the historical use of these three (Kozera and Rapacz: doi:10.1007/s13353-013-0173-x, Chapman and Waldenstrom: doi:10.1371/journal.pone.0141853), which we feel more comprehensively addresses the pervasive use of these genes.

*Also, it would be great if you could provide a table (or just an extension of Table 1) with a brief description of the condition/scenario in which the gene was suggested/validated as the best reference gene and the corresponding reference. That would make your introduction really valuable.*

 This is an excellent suggestion, but perhaps one best suited to a review article examining the use of reference genes more generally (rather than one focussed on identification of the correct references in developing mouse embryos specifically). Some of these genes (18S, ActB and GAPDH in particular, as discussed in the citations added above) have been used broadly, in scenarios ranging from mammalian cell cultures (Liu et al, doi: 10.1371/journal.pone.0117058) all the way to celery (Feng et al, doi: 10.7717/peerj.7925): including such a breadth of information, even in table form, seems challenging.

 Additionally, historical use of these genes might not always have been appropriate: validation of specific genes for specific scenarios is scientifically rigorous, but is also a comparatively recent approach. Such validation is typically conducted *a priori* rather than with the assumption that behaviour of a given gene in one context will translate to another. Indeed, several genes from the geNorm/geNorm PLUS collections (which comprise the bulk of those used here) were selected not due to their use in the literature, but based on their stability across multiple publicly accessible microarray datasets: the core principle being that providing a large panel of candidate genes (all of which should ostensibly be relatively stable) allows users to determine the most stable genes for their specific scenario (as in this manuscript). 18S, GAPDH and ActB are typically included in geNorm collections not because they are likely to be stable, but for the simple reason that they have such broad historical use: their inclusion thus allows analysis to be placed in context with established literature.

 We already cite studies validating individual genes where appropriate (i.e. in neural or skeletal muscle contexts), and we are reluctant to further expand table 1 (which has already been enlarged to include accession numbers and amplicon lengths).

*Methods:*

*"Uterine horns were quickly removed and placed on ice (additional organs and muscles were collected from the adults for separate studies)."*

*I did not understand why this information is relevant to this study.*

 We apologise for any confusion: Wellcome Open Research adheres closely to the ARRIVE guidelines, and to the principles of Replacement, Reduction and Refinement (3Rs) -we feel this is laudable, and as a Veterinary College, we too adhere closely to these principles. This information was included to clarify explicitly that tissues from animals sacrificed for this work were utilised more broadly, both to demonstrate our commitment to reductions in animal use, and to encourage others to employ similar ethical approaches. We would prefer to retain this information for these reasons, but we are willing to remove it at the discretion of the editor.

*"RNA yield and purity were assessed via nanodrop (ND1000) and samples with 260/230 ratios below 1.7 were subjected to a second precipitation."*

*= > just for your information, the optimal values for RNA purity are: 260/280 = 2, and 260/230 = 2-2.2.*

 Thank you. We are aware of these optimal values, however we are also aware that it is not always possible to achieve such high ratios (particularly for 260/230), and that repeat cleaning of perfectly useable RNA in an attempt to attain greater purity is an approach with rapidly diminishing returns. In our experience, using the reagents cited in our manuscript, samples with 260/230 ratios above 1.7 behave essentially identically to those with optimal ratios, and this more modest threshold is markedly easier to attain. We feel this information might be of use/interest to other investigators.

*Make a table for all gene primer sequences and give the efficiency values for each.*

 As noted within the manuscript, sequences for most of the primers used in this work are proprietary, and thus cannot be provided. For the remaining six primers (as shown) we did not feel a table was necessary. The suggestion to show the efficiency values is excellent and appreciated, however: accordingly we have added all efficiency calculations and derived values to the extended data (see also our response to reviewer 1).

*Results:*

*Fig2. I am a little confused about this figure or maybe the legend is not clear enough. Are these points in each boxplot for all samples (i.e. all biological replicates for whole embryos, heads and forelimbs) per gene? Why is C shown without a box?*

 Yes, for each gene, all sample Cq values are shown (whole embryos, heads, forelimbs). Boxplots are depicted to more clearly illustrate the outlier behaviour of the specific samples identified as aberrant. Figure 2C serves exclusively to illustrate differences in specific sample batches within a single gene (18S or dp71), rather than overall consistency within a gene, and thus boxplots would not convey any further information beyond that already depicted. We accept that this might lead to confusion, however, and have thus altered this latter plot to box and whisker for consistency.

  We have also edited the figure legend to clarify these specifics (edited text in bold):

 “Box and whisker plots of **all** sample Cq values for each candidate gene used in this study: **each point represents the Cq value of an individual sample, for the gene indicated**. ( **A**)  Initial raw Cq values revealed a marked variance in 18S expression (but not other genes), restricted to samples from a single cDNA synthesis batch (open circles), compared to samples from other cDNA synthesis batches (black points), indicative of impaired random priming. ( **B**) Preparation of fresh cDNA for these samples corrected this discrepancy (filled circles), producing values consistent with other batches (black points). ( **C**) qPCR for 18S and the dystrophin isoform dp71 using the initial poor samples (1, **open circles**), the other samples (2, **black points)** and the fresh preparation (3, **filled circles**) shows that impaired random priming prevents accurate quantification of long transcripts. **Details of specific cDNA synthesis batches can be found in the underlying data** **^67^** **.**”

*Discussion:*

*It can be seen from Fig. 2 that the Ct values for the proposed reference genes are between 22 and 23, indicating that the genes are comparatively highly expressed. I would add a few lines to discuss whether you see a limitation in using these genes for the normalization of low expressed genes.*

**

 An excellent suggestion: thank you! We agree that it is preferable to use reference genes of comparable expression level to a given gene of interest, wherever practical. It can be challenging to identify stably expressed genes of low abundance, however (particularly within the transcriptionally dynamic tissue of embryos): low abundance is often an indicator of tightly controlled (rather than ubiquitous) expression, and moreover even if such genes are indeed constitutively expressed, lower levels of expression are inherently more vulnerable to stochastic noise. This can be partly addressed by use of multiple references (and is a strong argument in favour of such) but identifying *multiple* stably expressed low abundance genes is particularly challenging.

 Provided care is taken to ensure that efficiencies are appropriate and comparable between references and GOI, we feel that good, high abundance references are preferable to more noise-susceptible low abundance references, but we thank the reviewer for raising this excellent point, and have added words to address this within our discussion, as follows (new text in bold):

 “ **We note that all three genes are expressed at relatively high levels (Cq values of 21-24), potentially rendering them less appropriate for normalising low abundance transcripts (where minor differences in PCR efficiency can compound over multiple additional cycles). Identifying stably expressed low abundance genes can be challenging, however, and matching reference and GOI expression levels on a case-by-case basis is both time- and resource-intensive (and indeed impossible when investigating GOIs with dramatic changes in expression). Provided care is taken to use multiple stable, well-validated references (as per MIQE guidelines** **^24^** **), the utility of a broadly applicable high-abundance panel likely outweighs these concerns. Accordingly**, our validation process confirmed the utility of these genes, and also revealed insight into changes in gene expression during development…”

*I would also add a few lines emphasizing that the proposed reference genes are uniformly expressed throughout embryonic development, whereas the choice of these genes might be different if we were looking at individual parts (e.g., head, forelimbs) or* *scenarios.*

 A sensible suggestion: we agree. We have endeavoured to make these distinctions clear throughout our manuscript, and to stress that the three candidates identified are in fact not only highest scoring in whole embryos, but also within heads, and within the entire dataset (including whole embryos, heads and forelimbs). As noted above (and within the manuscript) they are not the *highest* scoring within forelimbs, but remain *high* scoring (such that data normalised using these three is comparable to data normalised using 18S/EIF4A. Given the reviewer’s suggestions, we have made changes to better clarify this (and to stress that such universality should not be assumed to extrapolate to additional scenarios). We have edited the text of the discussion and conclusions accordingly:

 “We note **these findings do not imply that these genes will necessarily be appropriate for more focussed embryonic contexts (such as specific organs), and suitable reference genes might need to be independently validated for such specific scenarios: indeed, as shown here** *18S* and *EIF4A* apparently represent better candidates in forelimbs specifically **(though *AP3D1*, *RPL13A* and *PAK1IP1* also scored highly)**. **S**tudies addressing developmental changes in limbs alone might technically be better served by these two, but this would necessarily sacrifice the utility advantages offered by a universal panel…”

 “Our data suggests that normalisation of expression over this period is not only possible, but that many reference genes are acceptable, though the genes *AP3D1*, *RPL13A* and *PAK1IP1* are the strongest candidates overall **(using the entire sample set), within whole embryos alone, or within heads. These genes are also strong candidates for use in forelimbs (though *18S* and *EIF4A* score fractionally higher in this tissue).** Our data **further** suggests only a pair of genes is necessary for effective normalisation: *AP3D1* and one other.”

# Peer review: Reviewer response for version 1

**DOI:** 10.21956/wellcomeopenres.18735.r45974

**Published:** 2021-10-21

**Authors:** Caroline L. Smith

**Affiliations:** 1 School of Life Sciences, University of Westminster, London, UK

This is a valuable resource for researchers which provides details of reference genes at different stages of embryonic development. As the authors comment, incorrect selection of reference genes can significantly skew qPCR data and furthermore some genes considered to be stable do change throughout development. The reports developmental regulation of about HPRT1 and B2M are valuable findings.

 Although the authors state that primer efficiencies were all 95-105%, it would be extremely helpful to have these values in a table alongside details of range of dilutions of cDNA used to generate these standard curves and which ages/tissues were used to determine the efficiencies.

 The primer sequences for the GeNorm are not available but it would be useful to know the amplicon sizes and accession numbers of the genes provided in Table 1.

 In Figure 2 - the legend lacks detail about which ages/tissues are used in the cDNA batches.

Is the work clearly and accurately presented and does it cite the current literature?

Yes

If applicable, is the statistical analysis and its interpretation appropriate?

Yes

Are all the source data underlying the results available to ensure full reproducibility?

Yes

Is the study design appropriate and is the work technically sound?

Yes

Are the conclusions drawn adequately supported by the results?

Yes

Are sufficient details of methods and analysis provided to allow replication by others?

Yes

Reviewer Expertise:

Gene expression, cardiovascular signalling

I confirm that I have read this submission and believe that I have an appropriate level of expertise to confirm that it is of an acceptable scientific standard.

# Response

**Published:** 2022-09-12

**Authors:** John Hildyard

We thank the reviewer for their supportive assessment of this work, and for the excellent suggestions (repeated below in italics, for reference), which we address as follows.

*Although the authors state that primer efficiencies were all 95-105%, it would be extremely helpful to have these values in a table alongside details of range of dilutions of cDNA used to generate these standard curves and which ages/tissues were used to determine the efficiencies*

 This is a pertinent and sensible suggestion: we have added two supplementary data files (extended data, Additional reference gene validation) containing all the efficiency calculations and the methodology used to generate these.

*The primer sequences for the GeNorm are not available but it would be useful to know the amplicon sizes and accession numbers of the genes provided in Table 1*

 Again, an excellent suggestion: we have added these details to the table.

*In Figure 2 - the legend lacks detail about which ages/tissues are used in the cDNA batches.*

 Correct, it did not: the primary purpose of figure 2 is to illustrate that batch-specific effects can be identified and then subsequently addressed (i.e. batch-specific behaviour eliminated), and thus for all subsequent analyses within the manuscript, no such batch effects should be assumed to apply. We did not consequently feel this additional information was necessarily of benefit to the reader, however we are entirely happy to implement this change as suggested. Accordingly, we have edited the legend to refer to the underlying data, which now contains the relevant batch-specific information (edits in bold).

 “Box and whisker plots of **all** sample Cq values for each candidate gene used in this study: **each point represents the Cq value of an individual sample, for the gene indicated**. ( **A**)  Initial raw Cq values revealed a marked variance in 18S expression (but not other genes), restricted to samples from a single cDNA synthesis batch (open circles), compared to samples from other cDNA synthesis batches (black points), indicative of impaired random priming. ( **B**) Preparation of fresh cDNA for these samples corrected this discrepancy (filled circles), producing values consistent with other batches (black points). ( **C**) qPCR for 18S and the dystrophin isoform dp71 using the initial poor samples (1, **open circles**), the other samples (2, **black points)** and the fresh preparation (3, **filled circles**) shows that impaired random priming prevents accurate quantification of long transcripts. **Details of specific cDNA synthesis batches can be found in the underlying data** **^67^** **.**”
